# Campaigning for science and scientists

**DOI:** 10.7554/eLife.106701

**Published:** 2025-03-27

**Authors:** Adriana Bankston

**Affiliations:** 1 ASGCT-AAAS Congressional Policy Fellow Washington, DC United States

**Keywords:** Science Under Threat in the United States, science, politics, science funding, science policy, careers in science, basic research, None

## Abstract

A Congressional Science & Technology policy fellow outlines some options available for responding to the blatant attacks on science and the scientific workforce in the US.

Since President Trump took office, we have seen a complete disregard for science and technology. The new administration’s attacks on science will severely harm our nation’s competitiveness and gravely weaken and diminish its scientific workforce, with researchers from underrepresented groups being particularly at risk.

At the federal agency level, we have seen grant reviews being delayed at the National Institutes of Health and other agencies, funds for scientific research being cut, web pages about federal grants being taken down, and loyal and dedicated workers being fired regardless of career stage. Firing federal workers will delay both current federal grant funding and future grant timelines, hindering our nation’s ability to sustain research discovery and develop cures for diseases. As a result, many researchers are now looking for careers in other sectors. This is particularly true for the many graduate students and postdoctoral researchers who use grant funding to pay for bills and support their families.

At the university level, scientific research is being hindered by a lack of funds for equipment, reagents and supplies needed to perform wet lab work (and the hardware and software needed for computational work). The actions of the new administration have also messed up grant writing and funding cycles, which will severely harm and disrupt on-going lines of research, and make it even harder for early-career scientists to contribute to the research workforce and to US competitiveness in science and technology. This is particularly true for women, minorities and other populations who are underrepresented in science, as many of the schemes and mechanisms introduced to increase the representation of such groups across scientific fields have been discontinued.

## How individual scientists can respond

Since these attacks on science have begun, one silver lining has been the wave of activism and speaking up that we have seen from scientists themselves. Several researchers have shared their experiences and articulated how these moves will be devastating for the scientific enterprise and future generations who will be unable to pursue research careers due to lack of funds. Many early-career researchers have spoken out on the impacts of the Trump administration’s actions on research opportunities and future careers in science, which is a testament to their bravery. It is also important that those who can express their views do so because many scientists are unable to speak up – or are too scared to for fear of consequences. Speaking up also helps researchers come together as a community.

Additionally, the Stand Up for Science rallies held across the country and the world have been an inspiring way to voice and clarify why science matters, and for the general public to see that scientists are regular people. Demonstrations like these also allow individuals from multiple groups to come together and defend science and scientists, including federal agency leaders, Members of Congress, activists, and researchers themselves.

These measures to defend science are important, but they need not stop here. Scientists should continue to contact their elected officials in multiple branches of government, including the US Congress. For students and trainees reading this, don’t give up on your determination to do good science because tackling our society’s challenges will require discoveries in many areas of research. And for scientists who would like to join the effort of influencing policy for science from a higher level, please consider applying for a Congressional policy opportunity, like the fellowship held by the author.

## How universities can respond

The actions of the new administration are also placing universities under pressure, and it will not be surprising if universities admit fewer graduate students and postdoctoral researchers, stop job interviews, and rescind research job offers that have already been made, all of which have already happened to an extent. This wave of federal research funding cuts will inevitably drive many early-career researchers to look for jobs in other sectors, and will cause potential future scientists (including trainees who may have been interested in a career in research) to reconsider.

However, there are a number of actions that universities could take. They could identify contacts at federal agencies who can help them navigate the current funding landscape. They could identify additional funding sources to help support salaries and benefits for trainees. And they could find alternative grants to support women, minorities, and other populations underrepresented in science to address the fact that diversity programs at federal agencies are being closed down and access to those funds has been blocked in many cases. This last point is important in order to ensure that the scientific workforce in the US reflects the diversity of the American population, and that scientists from different backgrounds can see themselves having careers in research for the long-term. One solution that could help is strengthening partnerships between R1 universities and R2 schools, including historically black colleges and universities, minority-serving institutions, and emerging research institutions. These measures could help conserve resources and better support the scientific workforce more broadly across the nation.

Finally, universities are part of a larger ecosystem that is concerned with scientific innovation. I would encourage researchers – from early-career trainees to senior scientists – and university coalitions and affinity groups to continue pushing Congress to hold President Trump and his administration accountable for their detrimental actions on science. In particular, we need to know what the new administration will do to address the “brain drain” from research in the US, and make it clear to them how these actions will harm our nation’s ability to lead in research and develop a strong scientific workforce.

